# A decision rule to aid selection of patients with abdominal sepsis requiring a relaparotomy

**DOI:** 10.1186/1471-2482-13-28

**Published:** 2013-07-19

**Authors:** Jordy JS Kiewiet, Oddeke van Ruler, Marja A Boermeester, Johannes B Reitsma

**Affiliations:** 1 Department of Surgery, Academic Medical Center, Meibergdreef 9, Amsterdam 1105 AZ, The Netherlands; 2 Department of Clinical Epidemiology and Biostatistics, Academic Medical Center, Amsterdam, The Netherlands

**Keywords:** Secondary peritonitis, Abdominal sepsis, Relaparotomy, On-demand, Prediction model, Decision rule

## Abstract

**Background:**

Accurate and timely identification of patients in need of a relaparotomy is challenging since there are no readily available strongholds. The aim of this study is to develop a prediction model to aid the decision-making process in whom to perform a relaparotomy.

**Methods:**

Data from a randomized trial comparing surgical strategies for relaparotomy were used. Variables were selected based on previous reports and common clinical sense and screened in a univariable regression analysis to identify those associated with the need for relaparotomy. Variables with the strongest association were considered for the prediction model which was constructed after backward elimination in a multivariable regression analysis. The discriminatory capacity of the model was expressed with the area under the curve (AUC). A cut-off analysis was performed to illustrate the consequences in clinical practice.

**Results:**

One hundred and eighty-two patients were included; 46 were considered cases requiring a relaparotomy. A prediction model was build containing 6 variables. This final model had an AUC of 0.80 indicating good discriminatory capacity. However, acceptable sensitivity would require a low threshold for relaparotomy leading to an unacceptable rate of negative relaparotomies (63%). Therefore, the prediction model was incorporated in a decision rule were the interval until re-assessment and the use of Computed Tomography are related to the outcome of the model.

**Conclusions:**

To construct a prediction model that will provide a definite answer whether or not to perform a relaparotomy seems a utopia. However, our prediction model can be used to stratify patients on their underlying risk and could guide further monitoring of patients with abdominal sepsis in order to identify patients with suspected ongoing peritonitis in a timely fashion.

## Background

Secondary peritonitis is a frequently encountered entity in general surgical practice [1]. An estimation of the annual incidence is difficult, as the underlying causes of peritonitis are diverse (e.g. gastric ulcer perforation, complicated diverticulitis). Because of this diversity, the initial surgical approach differs and uniformity of treatment is hard to achieve. After the initial laparotomy a common challenge arises [2-5]. The challenge being whether or not there is ongoing peritonitis, and whether or not a relaparotomy is necessary. Several surgical strategies have been introduced in an attempt to reduce the high mortality accompanying severe secondary peritonitis [6].

Recently our study group reported the results of a randomized controlled trial comparing the two most frequently used surgical strategies after the initial laparotomy, namely relaparotomy on demand and planned relaparotomy [7]. In the on demand strategy a relaparotomy is performed only when there is clinical deterioration or lack of improvement, whereas in the planned strategy a relaparotomy is planned every 48 hours unless findings at relaparotomy are negative. On-demand led to a non-significantly lower mortality and morbidity compared to planned. However, other endpoints such as Intensive Care Unit (ICU) stay, duration of mechanical ventilation, number of interventions, and costs were significantly in favor of relaparotomy on-demand. These results in favor of the on-demand strategy were also present for the most severely ill patients with initial APACHE-II scores of more than 20. Further analysis showed that the on-demand strategy is also favorable for patients with diffuse fecal peritonitis. Thus, these patients can be treated safe and effective with the on-demand strategy, and do not need traditional planned relaparotomy. Furthermore, when using the on-demand strategy one in three relaparotomies is negative whereas two out of three relaparotomies are negative when the planned strategy is used [7].

Vigilant monitoring of the patient is a requirement in the on-demand strategy [8]. Round the clock decision-making in order to perform a timely reoperation will improve the clinical outcomes of this strategy. In current practice, there is no consensus on what specific signs, symptoms, or lab values the decision to perform a relaparotomy should be based [6,9,10]. Every involved professional will agree that the identification of these parameters is not straightforward. A previous report suggests that variables indicative for persistent organ failure during early postoperative follow-up are the best predictors [11]. An extensive model with 16 variables was constructed in this study limiting its use in clinical practice. Elaborating on the findings of this study the aim of the present study is to use prospectively collected data of the before mentioned randomized trial to develop a prediction model to use in the decision-making process to identify patients in whom a relaparotomy is required.

## Methods

### Patients

This study is based on the randomized controlled trial (RELAP trial) comparing two surgical strategies in patients with secondary peritonitis after their initial emergency laparotomy [7]. The study was approved by the medical ethics committee of the Academic Medical Center, Amsterdam, the Netherlands and all participating hospitals. Inclusion criteria were an APACHE-II score of more then 10, age between 18 and 80, and written informed consent. Exclusion criteria were peritonitis due to perforation of the bowel after endoscopy operated within 24 hours, abdominal infection due to an indwelling peritoneal dialysis catheter, peritonitis caused by pancreatitis, expected survival of less than six months due to disseminated malignancy, severe brain damage due to trauma or anoxia, and residual surgical therapy needed to eliminate the primary intra-abdominal focus (e.g. temporary end-stapled bowel loops in case of ischemia, temporary packing with gauzes in case of severe bleeding during surgery, and proximal enterostomy without initial resection of a more distally located focus). The trial cohort consisted of 229 patients, 114 allocated to the on-demand strategy arm and 115 to the planned strategy arm. In the present study, patients from both treatment arms were included.

The outcome of the present study was the necessity of relaparotomy. This was based on the findings at relaparotomy in patients who underwent a relaparotomy on day two or three. In the present study, patients were included only if they underwent a relaparotomy on day two or three, or if they did not have a relaparotomy at all. Patients who had a relaparotomy in a later stage of the disease were excluded as such patients would blur the relation between the variable and its predictive value. In the RELAP trial 75% of patients with a relaparotomy underwent the procedure on day two or three, with the median timing on day two (range day 0–34).

Cases were patients “requiring a relaparotomy” consisting of patients who had positive findings at relaparotomy, indicating that relaparotomy was indeed necessary. Controls were patients “not requiring a relaparotomy” consisting of (a) patients in whom a relaparotomy was performed but findings were negative (i.e. no ongoing peritonitis) and (b) patients who recovered without the need of a relaparotomy. Exceptions were patients without a relaparotomy who died within 14 days after the initial laparotomy. Such early deaths without relaparotomy may indicate to different situations, including patients that were in need for a relaparotomy, but the diagnosis was missed, death from an unrelated condition, or abstention (restriction) of treatment. Because of this clinical and diagnostic uncertainty with lack of visual verification, these early deaths were excluded from the present study. Differences in baseline characteristics between cases and controls were analyzed with Mann–Whitney U tests and Chi square tests.

Data of the second day after the initial laparotomy was used to construct the prediction model for several reasons. For a start, the decision to perform a relaparotomy is most challenging in the first few days after the initial laparotomy. Furthermore, the variables of day one are likely to be influenced by the initial laparotomy and the variables of day two are closest in time to the median time when a relaparotomy was performed in the RELAP trial.

### Missing data

The amount of variables that had to be recorded was extensive and missing of data was inevitable. Although the missing data rate per variable was low (median 5%, range 0%-14%), it could create uncertainty or even bias in the logistic regression analysis when patients with missing data would have been excluded from the analysis. Therefore, we used multiple imputations in five rounds to replace missing values with a set of plausible variables that represented the uncertainty about the right value to impute [12,13]. All potential predictors in addition to the outcome variable were included in the imputation model. Imputation was done on the original continuous measurement scale of the predictors based on multivariate normal distributions [14]. For patients not admitted to the Intensive Care Unit (ICU), normal values were imputed for variables associated with ICU care (e.g. air oxygen pressure (FiO2) and central venous pressure (CVP)).

### Candidate variables and model building strategy

Model building and variable selection was done in three steps

Step 1: Selection of candidate variables. A total of 76 variables had been registered during the first 14 days after the initial laparotomy and were available for predictive statistical analysis. Thirty-two candidate variables were selected out of these 76 variables based on their potential predictive value and clinical applicability using clinical reasoning, common sense and previous reports [11,15] (Additional file 1).

Step 2: Univariable association of candidate variables. Variables that were selected in step 1 were assessed for their univariable association with necessary relaparotomy in a logistic regression analysis. Each variable that produced a p-value of <0.2 was passed to the multivariate analysis.

Step 3: Reduction of multivariable model. The final prediction model was build using the multivariable backward elimination logistic regression method successively dropping those variables with a multivariable p-value above 0.2. The sensitivity and specificity of the prediction model for various cut-off values of the risk score were plotted in a receiver operator characteristics (ROC) curve. The area under the curve (AUC) value with 95% confidence intervals (CI) was determined to express the discriminatory capacity. All reported confidence intervals are based on five rounds of imputation. To correct for over fitting of the prediction model a bootstrap analysis was performed. New areas under the curve, adjusted based on 500 bootstrap samples were calculated and a shrinkage factor was applied to the final prediction model.

### Impact of treatment

The assigned treatment (planned or on-demand) in the randomized trial could be a contributing variable in the need for a relaparotomy. To examine this, a logistic regression model was build containing the surgical strategy, the score for each patient calculated with the final prediction model, and an interaction term between these two. This interaction term can be used to evaluate whether the predictive capability of variables was comparable in both arms.

### Nomogram and decision rule

A nomogram was made of the final prediction model. In this nomogram the regression coefficients form the final multivariable logistic regression model were translated into points for each variable. The total score in points correlates with a absolute probability (expressed as a percentage) that a relaparotomy is necessary. To further enhance the clinical applicability of the nomogram three categories are distinguished: low, intermediate and high risk that a patient needs a relaparotomy. With these categories a decision rule was created acting as guideline in the monitoring of patients with abdominal sepsis.

### Cut-off analysis

To provide further insight in potential clinical consequences when using the final model to determine whether or not a patient should have a relaparotomy, we set specific cut-off values corresponding with the probability categories of the nomogram and decision rule. The upper border of the low risk category was considered the threshold not to perform a relaparotomy. The most serious consequence of not performing a relaparotomy is that patients are wrongfully withheld from the operation which is quantified in the negative predictive value (NPV), The NPV represents the reliability that a patient in the low probability category is truly not in need of a relaparotomy. The lower border of the high risk category was considered the threshold to perform a relaparotomy. When a relaparotomy is performed based on this cut-off value there are two clinically relevant questions; (a) how many patients do not receive a relaparotomy whilst needing one and (b) how many patients receive an unnecessary relaparotomy. These two questions are addressed by calculating (a) the sensitivity and (b) the false positive rate (FPR).

All statistical analyses were performed using SPSS® software (version 16) for Windows (SPSS, Chicago, Il, USA) and SAS® ¬¬software (version 9.1) for Windows (SAS Institute, Cary, NC, USA).

## Results

### Patients

**Figure 1:**
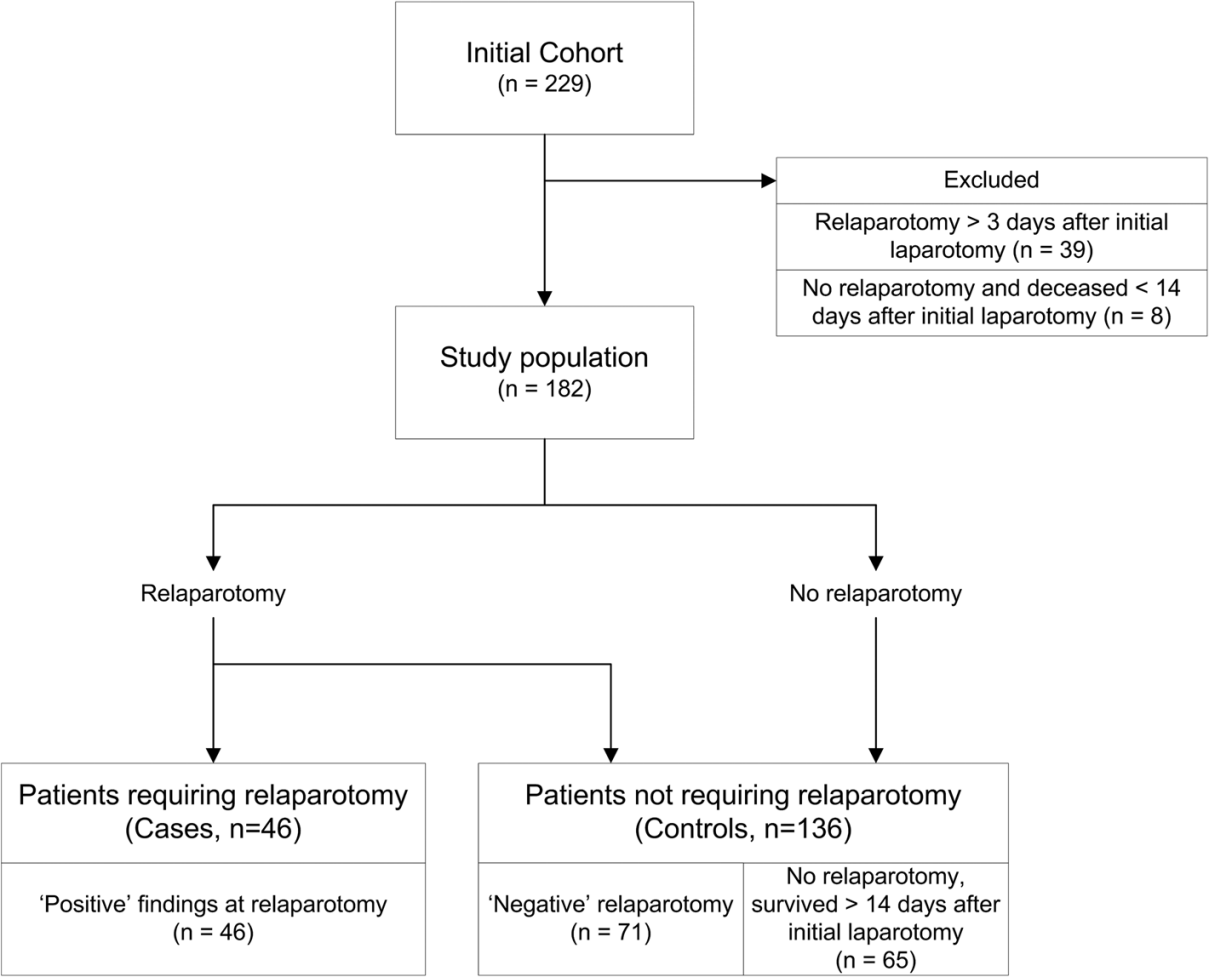
Inclusion of patients and outcome definition in the prediction study of patients requiring a relaparotomy.

Of the 229 patients randomized a total of 182 patients where included in the present study. A flowchart of the inclusion is depicted in Figure 1. A total of 47 patients were excluded from the analysis because they had a relaparotomy more than three days after the initial laparotomy (n = 39), or they had no relaparotomy and deceased within 14 days after their initial laparotomy (n = 8). Of the 182 included patients in the analysis, 46 patients needed a relaparotomy and 136 did not. Demographic and outcome data are displayed in Table 1. Age and gender did not differ between the compared groups. However, as can be expected in patients with positive findings at relaparotomy indicating persistent peritonitis, the length of ICU admission and in-hospital mortality differed significantly from patients who did not need a relaparotomy.

**Table 1:** Demographic characteristics and clinical outcomes in patients that did (cases) or did not (controls) require a relaparotomy

**Characteristic**	**Cases**	**Controls**	**p-value**
	**(n = 46)**	**(n = 136)**	
Median age in years^a^	68 (57–73)	70 (57–76)	0.467
Male	52%	58%	0.262
ICU admission	96%	90%	0.267
Length of ICU stay in days^a^	14 (6–31)	6 (2–14)	<0.001
Length of index hospital stay in days^a^	45 (21–76)	28 (18–51)	0.104
In-hospital mortality	30%	13%	0.008
APACHE-II score on study entry^a^	15 (12–16)	15 (13–18)	0.341

^a^Age, length of ICU stay, length of hospital stay, and APACHE-II score on study entry are displayed as a median with the interquartile range (IQR).

### Selected variables

Of the 32 candidate variables, 11 variables had a p-value of < 0.2 in the univariable regression analysis. Three operative and peritonitis related variables were identified: the extent of contamination during the initial laparotomy, the underlying cause of peritonitis and the elimination of the infectious focus during initial laparotomy. The remaining nine variables were all post-operatively recorded variables. These can roughly be subdivided into three categories: organ function dependent factors, related to the gastrointestinal tract function, and related to the requirement of supportive medication or other supportive measures. These 11 variables were entered in the multivariable analysis. Odds ratio (OR) and the associated 95% confidence interval (CI) are displayed in Table 2.

**Table 2:** Odds ratios (OR) and 95% confidence intervals (CI) of the variables entered in the multivariable logistic regression and after backward selection to predict the need for a relaparotomy in patients with secondary peritonitis

**Variable**	**Full model**		**Backward elimination**^**a**^	
	**OR**	**95% CI**	**OR**	**95% CI**
Heart rate > 90 bpm	1.61	0.57-4.53	1.97	0.75-5.17
Central venous pressure^b^	1.01	0.92-1.10	Dropped	
Lactate^b,c^	1.22	0.59-2.52	Dropped	
Platelet count < 150 x 10^9^/L	1.51	0.58-3.91	Dropped	
Hemoglobin < 5.0 mmol/L (8.1 g/dl)	3.23	1.07-9.76	2.99	1.10-8.10
Temperature <35.5 or >39.0°C	2.51	0.86-7.34	2.50	0.95-6.56
Administration of inotropic agents	4.99	1.80-13.84	4.09	1.66-10.07
No defecation	5.86	1.93-17.75	4.35	1.55-12.16
Diffuse contamination at initial operation	1.90	0.74-4.84	2.15	0.91-5.09
Etiology of peritonitis				
- inflammation	5.20	0.51-53.47	Dropped	
- perforation	1.00	reference		
- ischemia/necrosis	0.32	0.03-3.45		
- anastomotic leakage	1.64	0.62-4.34		
Elimination of infectious focus at initial operation	2.98	0.49-18.17	Dropped	

^a^ Corrected for over fitting, values of final model.

^b^ Continues variable, OR per unit increase in lactate (mmol/L) and central venous pressure (mmHg).

^c^ Logarithmic transformation.

### Final model

**Figure 2:**
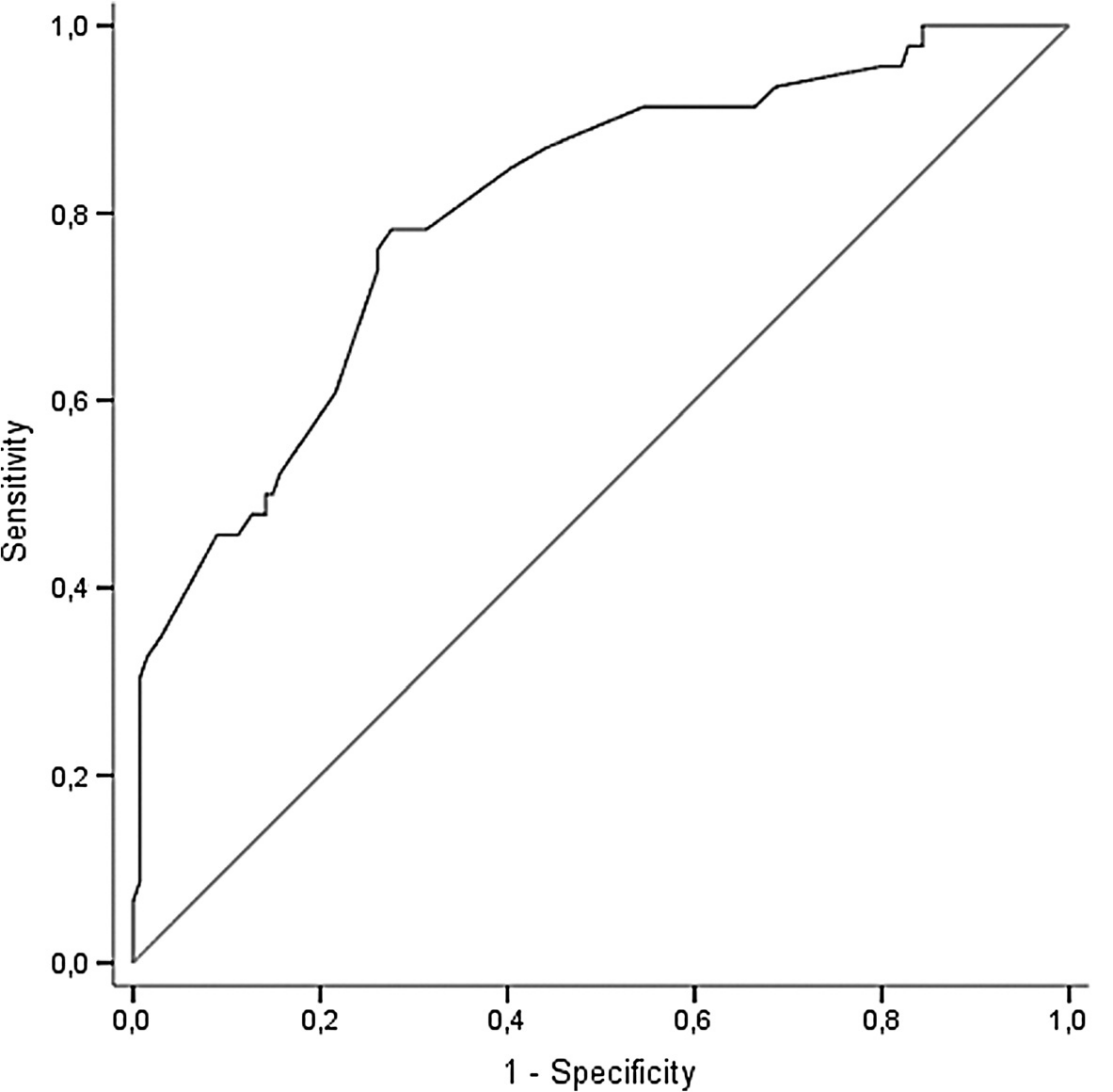
**Receiver operating characteristic (ROC) curve showing sensitivity and 1 minus specificity for various cut-off values of the risk score of the multivariable prediction model before adjustment for over fitting.** The area under this curve (AUC), a measure of discriminatory ability, is 0.83. After correction for over fitting the AUC is 0.80. The diagonal reference line indicates no discriminatory capacity (AUC 0.50).

After backward elimination, the following six variables remained in the final prediction model and were associated with an increased risk of needing relaparotomy: heart rate, hemoglobin level, body temperature, no defecation, the extent of the contamination found at the initial laparotomy, and the need for administration of inotropic agents. The discriminatory capacity of the final model had an area under the curve of 0.83 (range; 0.71-0.91). After adjustment for overfitting using bootstrap techniques the AUC is 0.80 (range; 0.69-0.82), suggesting reasonable discriminatory capacity Figure 2. In other words, the probability that a randomly chosen patient who needs a relaparotomy will have a higher score than a randomly chosen patient who does not need a relaparotomy is 80%. Table 2 displays the odds ratios and the 95% confidence intervals of the final prediction model after the shrinkage factor is applied.

### Treatment interaction

A significant p-value for the interaction term between the allocated surgical strategy and the risk score of the prediction model would indicate that the type of surgical strategy is of influence on the outcome of the analysis. The interaction term stated for the influence of the treatment (planned relaparotomy or relaparotomy on-demand), was not significant (p = 0.533). This indicates that the inclusion of patients from both treatment arms in the analysis did not influence the outcome of the risk prediction by the final model.

### Decision rule

**Figure 3:**
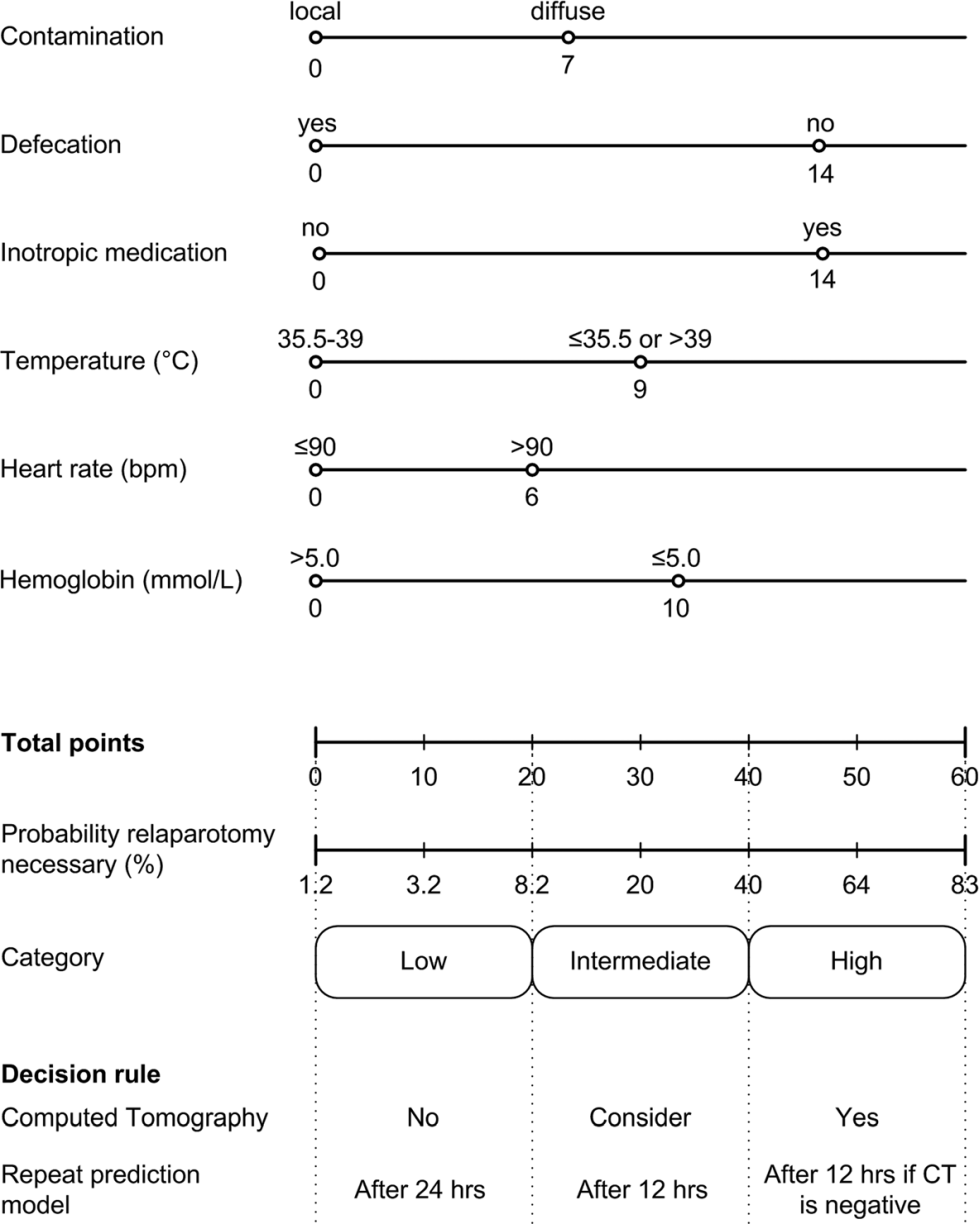
**Nomogram displaying the points associated with each variable included in the final prediction model corrected for over fitting.** The total score is converted to the probability that a relaparotomy is necessary and divided into three categories. The decision rule guides monitoring of the patient by timing the repetition of the prediction model and performance of a computed tomography scan if indicated.

The final prediction model is displayed as a nomogram in Figure 3. If a patient scores points on all six variables this would result in the maximum score possible of 60 points corresponding with an 83% probability that a relaparotomy is necessary. The outcome of the prediction model is divided into three categories on which the decisional rule is based. Patients in category one (less then 20 points) have a low predicted probability of needing a relaparotomy, and these patients are reassessed with the prediction model after 24 hours. For patients in the second category (20–40 points) with an intermediate probability, the prediction model is repeated after 12 hours and performing a CT in these patients can be considered. Patients in the last category (more then 40 points) have a high predicted probability indicating that an abdominal CT should be performed and if negative the prediction model should be repeated within 12 hours (Figure 3).

### Cut-off analysis

The cut-off value (after reduction and shrinkage) not to perform a relaparotomy was set at 20 points being the upper border of the low risk category. In this category 42 out of 45 patients are correctly identified not needing a relaparotomy resulting in a negative predictive value of 93% (Table 3). The NPV of the intermediate category (21–40 points) was 79%. The cut-off value to perform a relaparotomy was set at 40 points being the lower border of the high probability. Using this value in the final prediction model would result in the correct identification of 46% (21 of 46) of patients requiring a relaparotomy (sensitivity). When the cut-off value (of 40 points) would dictate relaparotomy, 34 patients would undergo a relaparotomy of which 13 would be unnecessary (FPR 42%). For this reason CT imaging was introduced in the decision rule’s third category.

**Table 3:** Performance of the final model at the cut-off values corresponding with the 3 categories of the decision rule

**Category**	**Relaparotomy necessary**	
	**Yes**	**No**
Low (score ≤ 20 )	3	42
Intermediate (score 21–40)	22	81
High (score > 40)	21	13

## Discussion

Identifying patients with ongoing peritonitis who are in need of a relaparotomy is challenging. This is endorsed by the results of the current prediction study. Although several clinical factors are associated with the need for relaparotomy with reasonable discriminative capacity, direct clinical decision-making based solely on the outcome of the prediction model is problematic. This is illustrated in the cut-off analysis were more then half of the patients in need of a relaparotomy will not receive a relaparotomy if the decision was based solely on the outcome of the prediction model. Using the threshold value of 40 points would lead negative relaparotomy rate of 42% which is certainly not an improvement compared to the 31% negative relaparotomy rate in the on-demand strategy in the RELAP trial. By lowering the threshold more patients in need of a relaparotomy would be identified correctly. However, to achieve a sensitivity of for instance 90% leads to an unacceptably high rate of unnecessary relaparotomies of 63% comparable to the 66% of the planned relaparotomy arm of the RELAP trial.

These findings suggest that the nomogram resulting from the prediction model cannot replace decision-making by the treating physicians. The outcome of the model merely provides a score that can be used in risk stratification. This means that the nomogram can be used to guide clinicians in the monitoring of the patient and what to do next. The prediction model is largely based on post-operative variables (5 out of 6). Therefore, repeating the prediction model is a standardized way of assessing the clinical situation. Decreasing the time interval between the repeated assessments in patients with higher predicted risks may therefore minimize the chance that a patient will not receive a relaparotomy when needing one. In addition the low-threshold use of CT in the higher risk categories could further improve the accuracy of decision-making whether or not to perform a relaparotomy. However, the additional diagnostic accuracy of CT should be investigated in future studies. The nomogram may be tested for use as a selection tool for CT evaluation in patients suspected of ongoing infection. The decision rule could improve the communication and transparency in decision making within the team while managing a complex and heterogeneous patient population. Especially since there are substantial differences between clinicians which variables they (subconsciously) use to base their decision on to perform a relaparotomy.

In the literature, several models have been developed and validated to predict mortality and morbidity in septic patients with secondary peritonitis [16-20]. However, none of these models have sufficient discriminatory capacity to predict the need for a relaparotomy [21]. These models incorporate some of the variables used in our prediction model but none of them includes more then one of these variables in the same model. There is only one prediction model, called the Abdominal Reoperation Predictive Index (ARPI), which has been designed specifically for the selection of patients for relaparotomy [22]. ARPI has limitations for use in clinical practice. First, the accuracy of this scoring system has never been reported, merely the relation between the score and the incidence of performing a relaparotomy. Second, the most heavily weighing variables (symptoms appearing from 4th day of surgery, the presence of a wound infection) are aimed at symptoms of a postoperative infectious complication after elective abdominal surgery, and not at the setting after surgery for peritonitis. Finally, subjective variables less appropriate for sedated and ventilated ICU patients, such as the presence of abdominal pain, weigh heavily. None of the variables included in the ARPI are part of the final model in the current study.

There are several limitations to the present study, because the data of the randomized trial were not collected for the specific use of constructing a prediction model. Patients who did not receive a relaparotomy but died within 14 days of the initial laparotomy were excluded. These patients could represent ‘cases’ in need of a relaparotomy, however the reasons why they where not operated remain unclear. To exclude patients who had a relaparotomy beyond three days after their initial operation, was an arbitrary decision. These patients also included ‘cases’ as some had positive findings during relaparotomy. Furthemore, the sample size is relatively small. The number of ‘cases’ is limited to 46 what could be of influence on the variance of the found predictive capacity. As a rule of thumb 10 cases should be included for every variable included in the final model. The course of secondary peritonitis, especially the first few days after the initial emergency laparotomy, can be complicated. Many events can occur which may need intervention [8]. To associate a variable with the need for a relaparotomy, the point in time when a variable is measured is important. The time between the measurement of a variable and the following relaparotomy must not be more than several hours. The events that can occur in a short period of time can have considerable influence on the measured value of a variable. To limit the influence that time has on the course of various parameters, and to enhance the homogeneity of the data, only data gathered on the second day after initial laparotomy were used. The data of the RELAP trial are not optimal to perform sequential statistical analysis with multiple observations in time. Therefore, the current analysis could not examine whether changes in values of predictive variables would improve the performance of the model. It is possible that worsening of values over time has more predictive value than the absolute value of that predictor. The allocation of the treatment arm could raise selection bias. Patients from both allocation arms were included in this study (planned relaparotomy 57% and on-demand 43%). Although there was a significant difference between allocation arms in the distribution of cases and controls (p < 0.05) the included treatment interaction analysis did not show significant influence on the prediction model. The constructed prediction model should be validated and, if needed, updated in a prospective study in which all potential predictors are measured at regular intervals in order to develop a more reliable model. This can be achieved with complete sequential recording a set of data, the relations with events taking place and the use of a decisional rule as proposed should be investigated.

## Conclusion

At present there are no clinically useful tools available to aid the decision-making process to select patients for a relaparotomy. This study illustrates that risk stratification of patients operated for abdominal sepsis using a nomogram could provide a clinical applicable tool. This tool might be used as a bed-side tool to aid in the timely selection of patients with ongoing peritonitis requiring relaparotomy.

## Competing interests

We wish to confirm that there are no known financial or non-financial conflicts of interest associated with this manuscript and there has been no significant financial support for this work that could have influenced its outcome.

## Authors’ contributions

JK participated in the study design, performed the statistical analysis and drafted the manuscript. OR collected the study data, participated in the study design and was involved in drafting of the manuscript. MB participated in the study design and critical revising of the manuscript. JB participated in the study design, performed the statistical analysis and critical revising of the manuscript. We confirm that the order of authors listed in the manuscript and the final version of the manuscript has been approved by all of us.

## Pre-publication history

The pre-publication history for this paper can be accessed here:

http://www.biomedcentral.com/1471-2482/13/28/prepub

## Supplementary Material

Additional file 1 Online supplementary information. Click here for file
