# Supplementary material for: A decision rule to aid selection of patients with abdominal sepsis requiring a relaparotomy
Source: BMC Surg. 2013 Jul 19;13:28. doi: 10.1186/1471-2482-13-28 (PMC3750491; doi:10.1186/1471-2482-13-28)
Supplement: Additional file 1 — Online supplementary information. [file 1471-2482-13-28-S1.doc]

**Appendix 1. Online supplementary information**

List of 76 variables recorded in the RELAP trial. Marked are the 32 selected candidate variables.

| **Patient related variables** | |
| --- | --- |
|  | Gender |
| ● | **Age** |
| ● | **Comorbidity present** |
|  | Major comorbidity present |
|  | History of major cardiac comorbidity |
|  | History of respiratory comorbidity |
|  | History of Diabetes Mellitus |
|  | History of renal disease |
|  | Malignancy present |
|  | Duration of symptoms until initial laparotomy |
|  | Abdominal sepsis after elective intestinal surgery |
|  |  |
| **Medication related variables** | |
| ● | **Administration of cardiac medication** |
|  | Administration of Epinephrine |
|  | Dose of Epinephrine |
|  | Administration of Norepinephrine |
|  | Dose of Norepinephrine |
|  | Administration of Dobutamine |
|  | Dose of Dobutamine |
|  | Administration of Ketanserin |
|  | Dose of Ketanserin |
|  | Administration of Enoximone |
|  | Dose of Enoximone |
|  | Administration of Dopamine |
|  | Dose of Dopamine |
|  | Administration of other inotropic medication |
|  | Dose of other inotropic medication |
|  | Administration of antibiotics |
|  | Change in use of antibiotics |
|  | Administration of Octreotide |
|  | Administration of corticosteroids |
|  |  |
| **Supportive treatment related variables** | |
|  | Mechanical ventilation |
|  | Renal replacement therapy |
|  | Use of fresh frozen plasma |
| ● | **Use of selective digestive decontamination** |
| ● | **Erythrocyte transfusion** |
|  |  |
| **Gastrointestinal tract function related variables** | |
|  | No administration of nutrition |
| ● | **Administration of enteral nutrition** |
|  | Adminstration of parental nutrition |
| ● | **Defecation** |
|  |  |
| **Initial laparotomy related variables** | |
|  | Primary anastomosis |
|  | Type of anastomosis in case of an anastomosis |
| ● | **Elimination of infectious focus** |
|  | Description of technique in case of elimination of infectious focus |
| ● | **Etiology of abdominal sepsis** |
| ● | **Localisation of etiology of abdominal sepsis** |
| ● | **Extent of contamination** |
| ● | **Type of contamination** |
|  | Primary closure of the abdomen |
|  |  |
|  |  |
| **Organ function related variables** | |
|  | Inspired oxygen fraction (FiO_2_) |
|  | Partial oxygen pressure (PaO_2_) |
|  | Systolic blood pressure |
|  | Diastolic blood pressure |
| ● | **Glasgow Coma Scale** |
| ● | **Heart rate** |
| ● | **Central venous pressure** |
| ● | **Respiratory rate** |
| ● | **Positive end expiratory pressure** |
| ● | **Urine production** |
| ● | **Temperature** |
| ● | **Mean arterial pressure** |
| ● | **PaO_2_/FiO_2_ ratio** |
|  |  |
| **Biochemical variables** | |
|  | Packed cell volume (Hematocrite) |
|  | Albumin |
|  | Activated partial thromboplastin time (aPTT) |
|  | Potassium |
| ● | **Creatinine** |
| ● | **Bilirubin** |
| ● | **Platelet count** |
| ● | **Potential of hydrogen (pH)** |
| ● | **Lactate** |
| ● | **Sodium** |
| ● | **Urea** |
| ● | **White blood cell count (WBC)** |
| ● | **Base excess (BE)** |
| ● | **C-reactive protein (CRP)** |
| ● | **Hemogoblin** |
